# Assessment of bone-implant interface image quality for in-vivo acetabular cup implants using photon-counting detector CT: Impact of tin pre-filtration

**DOI:** 10.1016/j.ejro.2025.100646

**Published:** 2025-03-27

**Authors:** Ronald Booij, Pauline de Klerk, Erik Tesselaar, Mischa Woisetschläger, Anne Brandts, Mariëlle Olsthoorn, Jakob van Oldenrijk, Koen Bos, Jörg Schilcher, Edwin H.G. Oei

**Affiliations:** a Department of Radiology & Nuclear Medicine, Erasmus Medical Center, Rotterdam, the Netherlands; b Center for Medical Image Science and Visualization (CMIV), Linköping University, Linköping, Sweden; c Department of Medical Radiation Physics, and Department of Health, Medicine and Caring Sciences, Linköping University, Linköping, Sweden; d Department of Radiology in Linköping and Department of Health, Medicine and Caring Sciences, Linköping University, Linköping, Sweden; e Department of Orthopedics and Sportsmedicine, Erasmus Medical Center, Rotterdam, the Netherlands; f Department of Orthopedics, Department of Clinical and Experimental Medicine, Linköping University, Linköping, Sweden; g Wallenberg Center for Molecular Medicine, Linköping University, Linköping, Sweden

**Keywords:** Arthroplasty, Image quality, Orthopedic Implants, Osseointegration, Photon-counting detector CT, -Tomography (x-ray computed)

## Abstract

**Purpose:**

To assess the image quality of the bone-implant interface of acetabular cup implants using photon-counting detector (PCD) CT with and without tin pre-filtration in a clinical setting.

**Methods and materials:**

Twenty-four patients underwent PCD-CT imaging of their total hip replacement (THR). Twelve patients were scanned using 140 kVp and twelve patients using 140 kVp with tin pre-filtration (Sn140 kVp). All scans were acquired with a collimation of 120 × 0.2 mm. The acquired data was reconstructed with different slice thickness (0.2 mm – 0.6 mm) and kernel (Qr) strengths (56, 76, 89) with and without metal artifact reduction (iMAR). Two observers assessed the image quality of the bone-implant interface for the cup based on four image quality criteria. Bone contrast, contrast-to-noise ratio (CNR) of bone/fat and cortical sharpness was performed as quantitative measures.

**Results:**

Image quality was rated highest for 0.2 mm slice thickness and Qr89 kernel across all four criteria for both the 140 kVp and Sn140 kVp by both observers, with a slight preference for the Sn140kVp over the 140 kVp. In all cases and for all image criteria the 0.2 mm/Qr89 was preferred above the Qr76 and Qr56/iMAR for both the 140 kVp and Sn140 kVp by both observers. Quantitative measurements confirmed significantly improved bone contrast as well as cortical sharpness using 0.2 mm/Qr89. Tin pre-filtration did not affect the CNR at 0.2 mm/Qr89 but tended to decrease cortical sharpness.

**Conclusions:**

High resolution PCD-CT allows for in-vivo assessment of the bone-implant interface in patients with THR and is preferably acquired with tin pre-filtration.

## 1 Introduction

The number of total joint replacements (TJRs) performed to treat end-stage osteoarthritis is rising [1]. In the United States alone, it is estimated that over 1.5 million first-time TJRs of the hip and knee will be conducted annually by 2030 [2]. Consequently, the number of patients seeking healthcare with a problematic TJR is likely to rise [3], [4]. Discrimination of implant related conditions from other differential diagnoses is important in the decision making to re-operate or not. The most common implant related complication after TJR is aseptic loosening [5], a condition where the integration of the implant into the surrounding bone tissue has failed. In our previous study, we demonstrated that PCD-CT has superior image quality regarding the assessment of osseointegration and visualization of the bone-implant interface in ex-vivo samples of acetabular cups compared to EID-CT [6]. As the implant hampers the visualization of the bone-implant interface, diagnostics of aseptic loosening remains challenging related to the effect of the implant on image quality causing metal artifacts [5]. However, tin pre-filtration at the tube site appears to be an effective method to reduce metal artifacts in energy-integrating detector (EID) CT and photon-counting detector (PCD) CT [7], [8]. In this study, we aimed to assess if PCD-CT with pre-filtration would be the preferred scan acquisition mode for the visualization of the bone-implant interface of acetabular cup implants in a clinical setting. Additionally, we aimed to assess the preferred reconstruction parameters for assessing an acetabular cup implant and highest diagnostic confidence for assessment of osseointegration.

## 2 Material and methods

### 2.1 Implants, image acquisition and reconstruction

#### 2.1.1 Data acquisition and implants

Between June 2021 and March 2023, all patients that underwent a clinically indicated PCD-CT (NAEOTOM Alpha, Siemens Healthineers, software version VA40) imaging of their hip and pelvis because of symptoms probably related to the total hip replacement (THR) were selected. During this period, two acquisition modes were used randomly in the clinical routine: the QUANTUM HD mode (120 ×0.2 mm slice collimation) with and without tin pre-filtration i.e. 140 kVp and Sn140 kVp, respectively. All patients were informed in accordance with the declaration of Helsinki and international standards of Good Clinical Practice. Additionally, the informed consent was signed accordingly. The choice for these two protocols was based on the need for high resolution and to investigate if the Sn140 kVp scan mode was providing improved image quality over the 140 kVp scan mode. Both modes were enabled with the ‘CARE keV’ dose optimized for ‘bone/calcium’. The image quality (IQ) level was set at 70 for both scan modes with a pitch of 0.4 and a rotation time of 0.5 seconds. Half of the 24 THR were scanned using 140 kVp and the other half with Sn140 kVp. Within the 140 kVp group, there were six cemented and six uncemented cups, while in the Sn140 kVp group there were eight cemented and four uncemented cups (Table 1).

**Table 1:** Type of acetabular cup implants and method of fixation.

kVp	cup cemented/uncemented	Cup Manufacturer
140	uncemented	Delta TT (Lima) Titanium
140	cemented	Charnley (Depuy) Ultra High Molecular Weight Polyethylene (UHMWPE)
140	uncemented	Pinnacle (Depuy)
140	uncemented	unkown
140	cemented	Avantage (Zimmer)
140	cemented	partial pelvis replacement shell (Link) + Avantage (Zimmer)
140	cemented	IP (Link)
140	uncemented	unkown
140	cemented	IP (Link)
140	uncemented	G7 (Zimmer)
140	uncemented	unkown
140	cemented	unkown

Sn140	cemented	partial pelvis replacement shell (Link) + Avantage (Zimmer)
Sn140	cemented	Marathon (Depuy)
Sn140	cemented	Charnley (Depuy)
Sn140	uncemented	Delta One (Lima)
Sn140	cemented	IP (Link)
Sn140	uncemented	unkown
Sn140	uncemented	unkown
Sn140	cemented	IP (Link)
Sn140	cemented	IP (Link)
Sn140	uncemented	Allofit (Zimmer)
Sn140	cemented	Stanmore (Zimmer)
Sn140	cemented	Stanmore (Zimmer)

#### 2.1.2 Image reconstruction

**Fig. 1:**
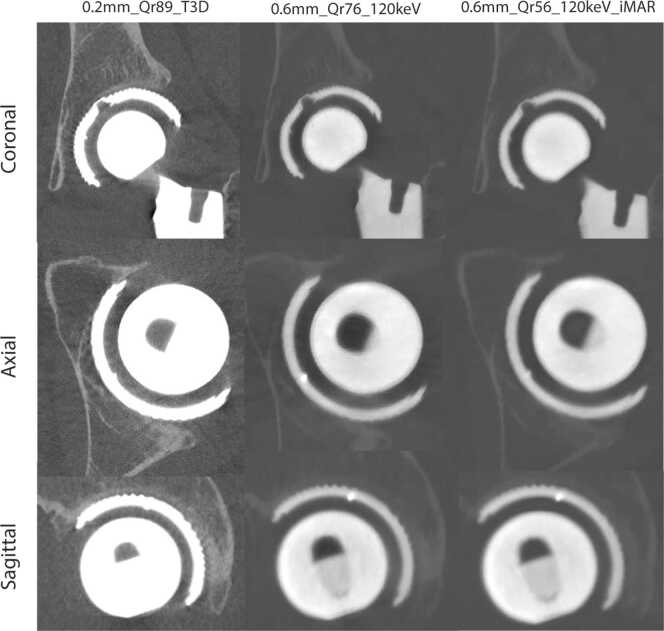
CT reconstructions used in the study showing an acetabular cup implant in different planes. The data was reconstructed in three ways: the left column shows the 0.2 mm Qr89 reconstructions, the middle column shows the 0.6 mm Qr76 and the column at the right shows the 0.6 mm Qr56_iMAR reconstructions. Window level and window center was 7500/2000 for all images.

In total, three image series were reconstructed with a slice thickness ranging from 0.2 mm – 0.6 mm with three different kernel strengths: with (Qr56) and without (Qr76 and Qr89) iterative metal artifact reduction (iMAR). The choice for these reconstructions were based on a previous study [6] and after a internally conducted preference study. For the reconstruction of the 0.2 mm slices (Qr89), a so called ‘T3D’ image was reconstructed, mimicking an energy level of 70 and 85 keV for the 140 kVp and Sn140 kVp, respectively. The 0.6 mm (kernels Qr56 and Qr76) were reconstructed as a virtual monoenergetic image (VMI) of 120 keV. Energy levels and reconstruction methods were based on literature outcomes and our previous study results, highlighting the preference of high kernels for osseointegration [6]. In addition to that, definite kernel and VMI level were chosen after local preference consensus meetings. Matrix size was automatically selected by the scanner software and were 768 × 768 for the Qr56 kernel and 1024 × 1024 for reconstructions with the Qr76 and Qr89 kernels to achieve the best spatial resolution in combination with field-of-view size, kernel type and strength. The iterative strength of the quantum iterative reconstruction (QIR) was set at 3. Fig. 1 demonstrate the different reconstruction types.

### 2.2 Image analysis

#### 2.2.1 Qualitative assessment

Two certified (three and seven years of experience) musculoskeletal radiologists were asked to review and grade the image series. They assessed the image quality of the bone-implant interface for the acetabular cup based on four image quality criteria using a 5-point Likert scale (from 1 to 5). The aim of the analysis was to obtain the preferred scan acquisition mode (with or without tin pre-filtration) and which one of the three reconstructions was preferred. The readers reviewed the series of images stacks blinded to the CT acquisition and reconstruction parameters. Images were anonymized and displayed in a random order on a clinical workstation using Syngo.via (Siemens Healthineers). The radiologists were allowed to zoom, pan, angle and change the window level/ window width settings. First, the observer was instructed to determine if the cup was cemented or uncemented. Accordingly, the review was conducted with four associated questions (Q1-Q4). Last, the observers were asked to provide their preferred reconstruction series per THR to assess the image quality of the bone-implant interface and highest diagnostic confidence for assessment of osseointegration. Criteria and rating scales for the assessment are presented in Table 2.

**Table 2:** Qualitative image quality criteria and rating scale.

Questions for the cemented cup:
1. There is a sharp delineation of the bone/cement interface
2. There is a sharp delineation of cement/poly interface
3. The image noise does not interfere with my clinical assessment
4. There is a sharp delineation of the trabecular bone structure

Questions for the uncemented cup:
1. There is a sharp delineation of the bone/implant interface
2. There is a sharp delineation of the titanium/poly interface
3. The image noise does not interfere with my clinical assessment
4. There is a sharp delineation of the trabecular bone structure

Answers in Likert scale:
1. I am sure that the criterion is not fulfilled: *'bad'*
2. I am almost sure that the criterion is not fulfilled: *'limited'*
3. I am not sure if the criterion is fulfilled or not: *'sufficient'*
4. I am almost sure that the criterion is fulfilled: *'good'*
5. I am sure that the criterion is fulfilled: *'excellent'*

#### 2.2.2 Quantitative assessment

As a good delineation of bone edges and visualization of anatomic relationships was of clinical importance for bone-implant assessment, the quantitative analysis included bone contrast, contrast-to-noise ratio (CNR) of bone/fat, and assessment of cortical sharpness. Bone contrast was defined as:

$${\mathrm{CNR}}_{\mathrm{bone}}=\;\frac{{\mathrm{CT}}_{\mathrm{bone}}-{\mathrm{CT}}_{\mathrm{fat}}}{{\mathrm{SD}}_{\mathrm{fat}}}$$

**Fig. 2:**
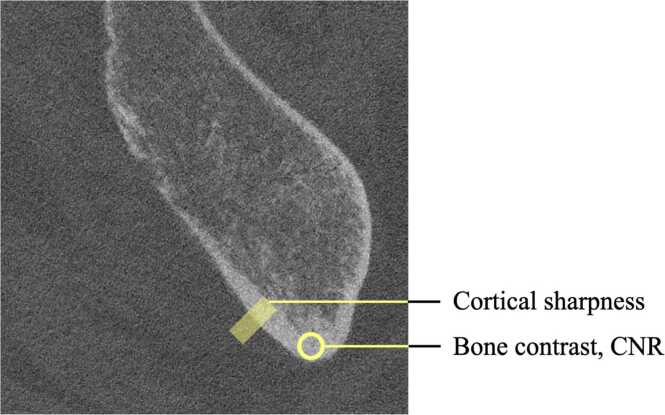
Positions of the measurements used to determine bone contrast, CNR of the bone/fat and the cortical sharpness.

Measurements were performed using Fiji v2.3.0/1.53 f [9]. Bone contrast and CNR were calculated as the difference between the mean CT number in circular regions of interest (diameter 5 mm) in cortical bone and in subcutaneous fat (Fig. 2). The width of the cortical edge was measured by fitting CT numbers along a line across the cortical edge to a mathematical error function and then estimating the full width half maximum (FWHM) as a measure of the cortical interface sharpness (in mm). This method has been described in detail previously [7].

### 2.3 Data analysis

Reader scores of the qualitative assessment were presented as median values as the data was non-normally distributed. The quantitative data of the cortical edge width, bone contrast and CNR of bone/fat were expressed as mean and standard deviation (SD) and were analyzed using mixed-effect analysis to test for differences between reconstruction kernels with or without tin pre-filtration. All analyses were done using GraphPad Prism version 9.0, (GraphPad Software). A p value below 0.05 was considered statistically significant.

## 3 Results

Twenty-four patients (nine men [age range 52–85 years] and fifteen women [age range 46 – 88 years]) were included after receiving a clinically indicated PCD-CT of their hips and pelvis because of symptoms probably related to the total hip replacement (THR).

### 3.1 Qualitative assessment

**Fig. 3:**
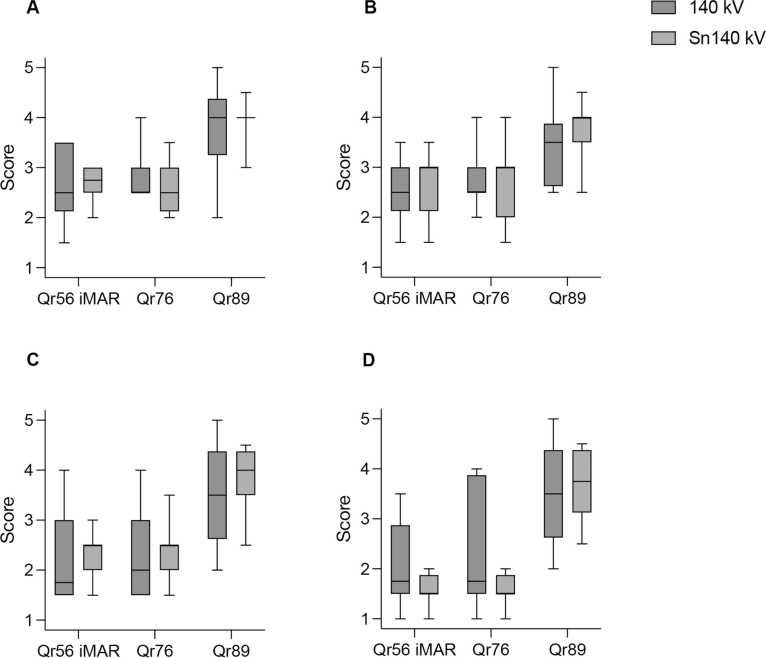
Reader scores for four different criteria (Q1 – Q4; where question/criteria Q1 =A; Q2 =B; Q3 =C and Q4 =D) for the two scan modes: Tube voltage of 140 kVp with and without tin pre-filtration; reconstruction kernels Qr89, Qr76 and Qr56 with iterative metal artifact reduction (iMAR). Boxes indicate interquartile range; whiskers indicate minimum and maximum values.

The median scores and interquartile range (IQR) of the two observers were taken per case and questions and is demonstrated in Fig. 3. Image quality was rated highest for 0.2 mm slice thickness and Qr89 kernel across all four criteria [median range Q1-Q4] for both the 140 kVp [3.5 – 4.0] and Sn140 kVp [4.0] by both radiologists. Reconstruction with 0.6 mm/Qr76 and 0.6 mm/Qr56/iMAR were rated with a median range of [1.75 – 2.5] for 140 kVp and [2.5 – 3.0] for Sn140 kVp. In all cases and for all image criteria the 0.2 mm/Qr89 was preferred above the Qr76 and Qr56/iMAR by both observers. The answers are based on analysis of all 24 cases (12 with and 12 without tin pre-filtration). Both observers rated the tin pre-filtration equal or superior in all cases, despite gender or fixation method. The observers indicated the 0.2 mm Qr89 series as their preference in 95 % of all cases and 5 % with no preference for the image quality assessment of the bone-implant interface of the acetabular cup. Both observers had a slight preference for the 0.2 mm/Qr89 reconstructions acquired with Sn140kVp over the 140 kVp demonstrated by the less spread of the ratings.

### 3.2 Quantitative assessment

**Fig. 4:**
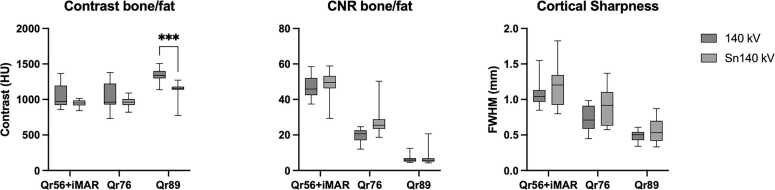
Results of the quantitative measurements, plotted per reconstruction and the difference between scan without (140kVp) and with (Sn140 kVp) tin pre-filtration.

The results of the quantitative measures are shown in Table 3 and Fig. 4. A sharper kernel strength significantly improved bone contrast (p < 0.0001) and reduced the cortical edge width (p < 0.001), although it reduced CNR (p < 0.0001) due to increased noise. Tin pre-filtration significantly reduced bone contrast, but only for Qr89 (p = 0.0002) and slightly reduced cortical sharpness, but not significantly (p = 0.11). The dose levels (CTDIvol) were on average 15.2 mGy and 13.9 mGy for 140 kVp and Sn140 kVp, respectively.

**Table 3:** Bone contrast, bone CNR and cortical sharpness for different reconstruction kernels and with and without tin pre-filtration.

		**Bone Contrast (HU)**	**Bone CNR**	**Cortical sharpness (mm)**
**140kVp**	0.6 mm Qr56 120 keV iMAR	1037 ± 168	47.3 ± 6.9	1.1 ± 0.21
	0.6 mm Qr76 120 keV	1036 ± 188	19.9 ± 3.5	0.73 ± 0.18
	0.2 mm Qr89 T3D	1340 ± 92	6.7 ± 2.6	0.49 ± 0.08

**Sn140kVp**	0.6 mm Qr56 120 keV iMAR	949 ± 47	48.8 ± 7.3	1.19 ± 0.31
	0.6 mm Qr76 120 keV	962 ± 70	27.4 ± 7.6	0.89 ± 0.27
	0.2 mm Qr89 T3D	1138 ± 113	7.2 ± 4.1	0.56 ± 0.17
Values are given as mean ± SD

## 4 Discussion

Assessing the bone-implant interface osseointegration to decide whether in implant has loosened from its bone bed is challenging. Based on previous reports, we hypothesized that PCD-CT with QUANTUM HD and tin pre-filtration might be the preferred scan mode for visualization of acetabular cup implants in-vivo. In our study, quantitative measurements showed significantly improved bone contrast as well as cortical sharpness using 0.2 mm/Qr89. Tin pre-filtration did not affect the CNR at 0.2 mm/Qr89 but tended to decrease cortical sharpness. The observers preferred PCD-CT with tin pre-filtration and reconstructed with thin slices, high kernel and high matrix and thin slices for the assessment of bone-implant interface.

Our results are in line with previous work from our group assessing the bone-implant interface in retrieved implants ex-vivo [6]. However, in that study we did not take into account the tin pre-filtration. Our study now addresses the possibilities for in-vivo assessment of an acetabular cup and potentially a direct assessment of osseointegration, especially when applying tin pre-filtration. Interestingly, the 0.2 mm images with high kernel and high matrix size had an overall higher score and preference, probably due to the sharper images offered and the decrease of metal artifacts due to the higher energy resulting from tin pre-filtration [10], [11], [12], [13]. A previous study also demonstrated that the use of tin pre-filtration is preferred [14]. In addition, they showed that a high keV VMI was preferred. However, only one kernel (Br76) and a slice thickness of 0.4 mm was used. Our study is a nice addition to this article, as we demonstrated that our observers had a strong preference for the higher kernel (Qr89) and thin slices (0.2 mm). This was demonstrated by the consistent scoring by both reader for the highest kernel (89), while there was less consistency between them for the lower kernels. Additionally, the additional tin filtration reduced metal artifacts and seemed more preferred than slightly reduced bone contrast. The implementation of tin-prefiltration and its impact on dose and image noise was also nicely highlighted in another study [15]. Overall, these results demonstrated here provide useful information in optimization of scan protocols with PCD-CT in clinical practice.

A recent paper addressed the use of metal artifact reduction software (iMAR) as well and concluded that this technique is preferred for reducing orthopedic implant artifacts [16]. However, this study primarily focused on reducing artifacts for the surrounding tissues, rather than the assessment of the implant itself. In addition, they didn’t use the highest resolution of 0.2 mm with high kernels (>56).

Our study has some limitations that need to be considered. First, the two different scan modes were applied in different patients, thus prohibiting any direct comparisons of the scan mode because we could not correct image quality for differences in body composition. Therefore, the small dose difference was not taken into account as a main determiner. Second, there are differences in fixation methods of how the acetabular cup was fixated (cemented or uncemented) and the number of patients were not equally distributed among the two scan modes. However, observers rated the tin pre-filtration equal or superior in all cases, despite gender or fixation method.

In conclusion, PCD-CT with the QUANTUM HD scan mode allows for adequate in-vivo assessment of the bone-implant interface. Tin pre-filtration seems preferred by radiologists. Ultimately, PCD-CT may be able to improve the assessment of osseointegration after joint replacement surgery. Therefore, PCD-CT may be valuable in discriminating a loosened THR as the source of pain from other differential diagnoses such as benign musculoskeletal conditions accordingly.

## Ethical approval

The Daily Board of the Medical Ethics Committee Erasmus MC (hereafter the Committee) of Rotterdam, The Netherlands, has declared that the rules laid down in the Medical Research Involving Human Subjects Act (also known by its Dutch abbreviation WMO), did not apply.

## Guarantor

The scientific guarantor of this publication is Ronald Booij.

## Statistics and Biometry

One of the authors has significant statistical expertise.

## Informed consent

All patients have given informed consent for collecting, using and storage of their data as described in the approved non-WMO-applicable research protocol ‘Photon-Counting Detector CT-scanner: A Prospective Data Registry’ (MEC-2021–0075).

## Methodology

• Observational
• Performed at one institution

## Funding

The authors state that this work has not received any funding.

## CRediT authorship contribution statement

**Tesselaar Erik:** Writing – review & editing, Writing – original draft, Validation, Software, Methodology, Formal analysis, Data curation, Conceptualization. **de Klerk Pauline:** Writing – review & editing, Validation, Investigation, Formal analysis, Data curation. **Booij Ronald:** Writing – review & editing, Writing – original draft, Visualization, Validation, Supervision, Software, Resources, Project administration, Methodology, Investigation, Formal analysis, Data curation, Conceptualization. **Oei Edwin:** Writing – review & editing, Writing – original draft, Validation, Supervision, Investigation, Conceptualization. **Schilcher Jörg:** Writing – review & editing, Writing – original draft, Conceptualization. **Bos Koen:** Writing – review & editing, Writing – original draft, Resources, Conceptualization. **van Oldenrijk Jakob:** Writing – review & editing, Writing – original draft, Resources, Conceptualization. **Olsthoorn Mariëlle:** Writing – review & editing, Writing – original draft, Methodology, Formal analysis. **Brandts Anne:** Writing – review & editing, Writing – original draft, Investigation, Formal analysis. **Woisetschläger Mischa:** Writing – review & editing, Writing – original draft, Conceptualization.

## Declaration of Competing Interest

The authors declare the following financial interests/personal relationships which may be considered as potential competing interests: The authors of this manuscript declare relationships with the following companies: CMIV and Erasmus MC receive institutional research support from Siemens Healthineers. If there are other authors, they declare that they have no known competing financial interests or personal relationships that could have appeared to influence the work reported in this paper.
